# Supplementary material for: Lattice-contraction triggered synchronous electrochromic actuator
Source: Nat Commun. 2018 Nov 15;9:4798. doi: 10.1038/s41467-018-07241-7 (PMC6237766; doi:10.1038/s41467-018-07241-7)
Supplement: Supplementary file 1 — Supplementary Information [file 41467_2018_7241_MOESM1_ESM.pdf]

Supplementary Information

Lattice-Contraction Triggered Synchronous Electrochromic Actuator

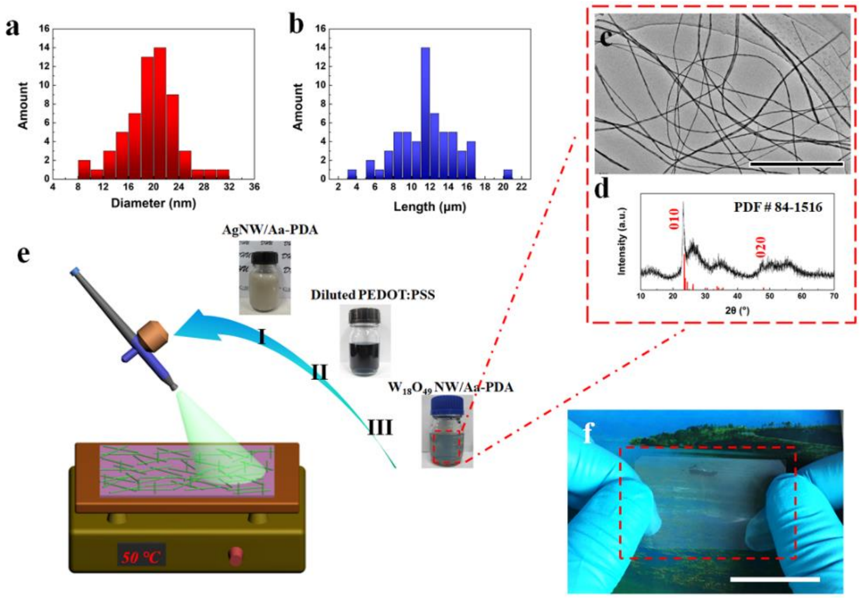

Supplementary Figure 1. The diameter (a) and length (b) distributions of $W_{18}O_{49}$NWs; (c) TEM images of $W_{18}O_{49}$NWs with low magnification (Scale bar: 1μm); (d) XRD of the as-obtained $W_{18}O_{49}$NWs; (e) Schematic illustration of the preparation of highly flexible dual-responsive films using a continuous spray-coating method; (f) A digital photograph of the dual-responsive film (Scale bar: 3 cm).

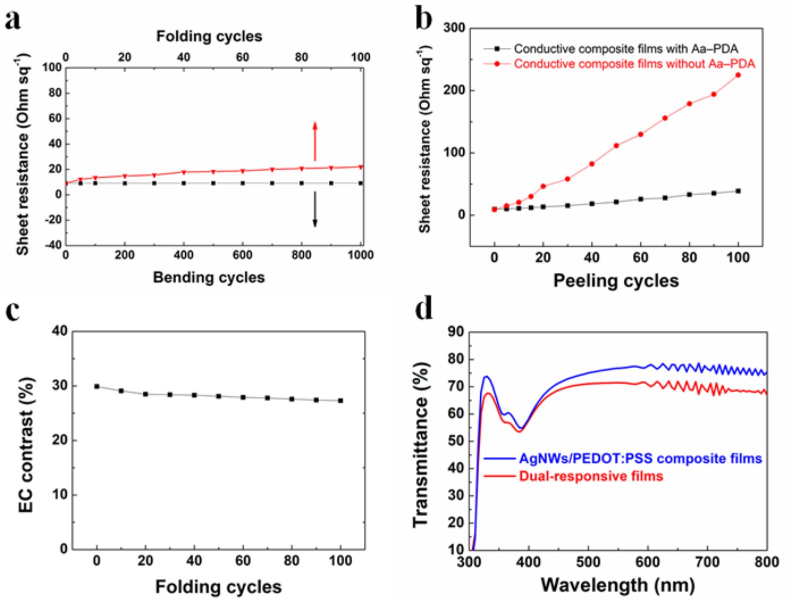

Supplementary Figure 2. (a) Sheet resistances as a function of bending and folding cycles for AgNWs/Aa-PDA/PEDOT:PSS conductive films (2.5 mm of bending radius of curvature); (b) Sheet resistances as a function of peeling cycles for the conductive composite films with/without Aa-PDA; (c) Optical contrasts of dual-responsive films as a function of folding cycles during 100 cycles; (d) Transmittance spectra of AgNWs/PEDOT:PSS composite films and dual-responsive films, respectively.

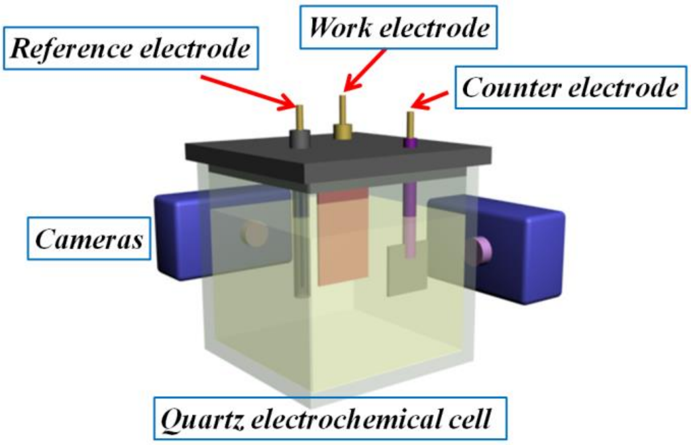

Supplementary Figure 3. Schematic illustration of *in situ* test equipment for electrochemical actuation measurement.

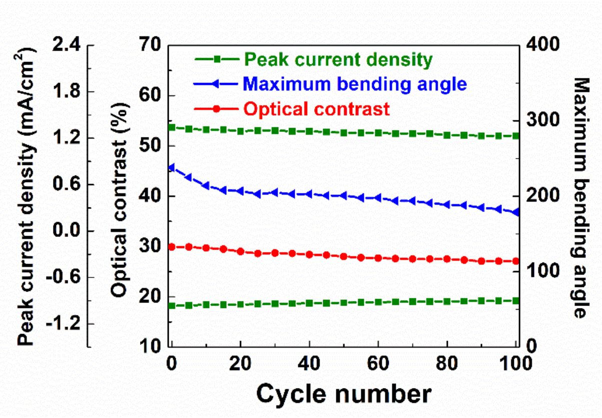

Supplementary Figure 4. Peak current density, optical contrast and maximum bending angle as a function of the step chronoamperometric cycles for dual-responsive films, respectively.

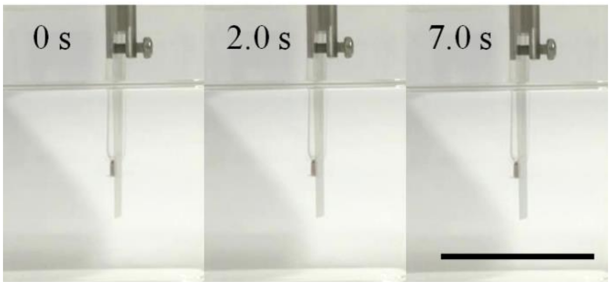

Supplementary Figure 5. Digital photographs of EC and deformation processes of the AgNWs/PEDOT:PSS composite film measured in 1M $LiClO_4$/PC electrolyte between +0.6 and –0.9 V bias (Scale bar: 4 cm).

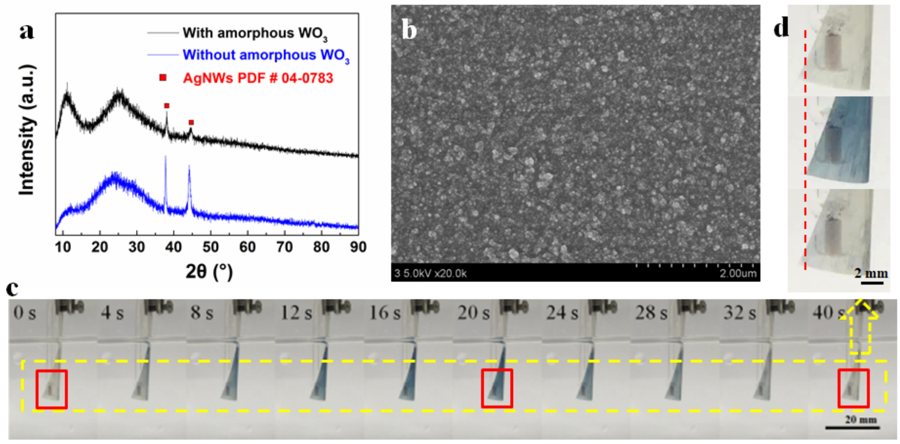

Supplementary Figure 6. (a) XRD curves of AgNWs/PEDOT:PSS composite films deposited with a-WO_3 and without a-WO_3, respectively; (b) SEM image of the AgNWs/PEDOT:PSS/a-WO_3 composite film; (c) Digital photographs of EC and deformation processes of the AgNWs/PEDOT:PSS/a-WO_3 composite film measured in 1 M LiClO4/PC; (d) Corresponding enlarged photographs of the deformation process.

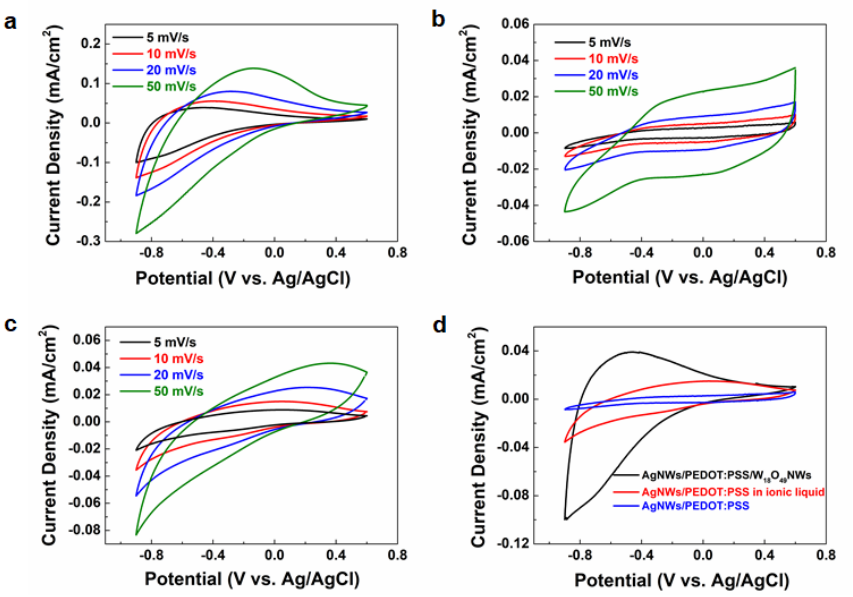

Supplementary Figure 7. Cyclic voltammetry (CV) curves of the dual-response films (a) and AgNWs/PEDOT:PSS composite films (b) measured in 1 M $LiClO_4$/PC electrolyte; (c) CV curves of the dual-response films measured in 1 M $EMIBF_4$/PC electrolyte; (d) The comparison among the respective CV curves (at scan rate of 5 mV/s) under above three conditions.

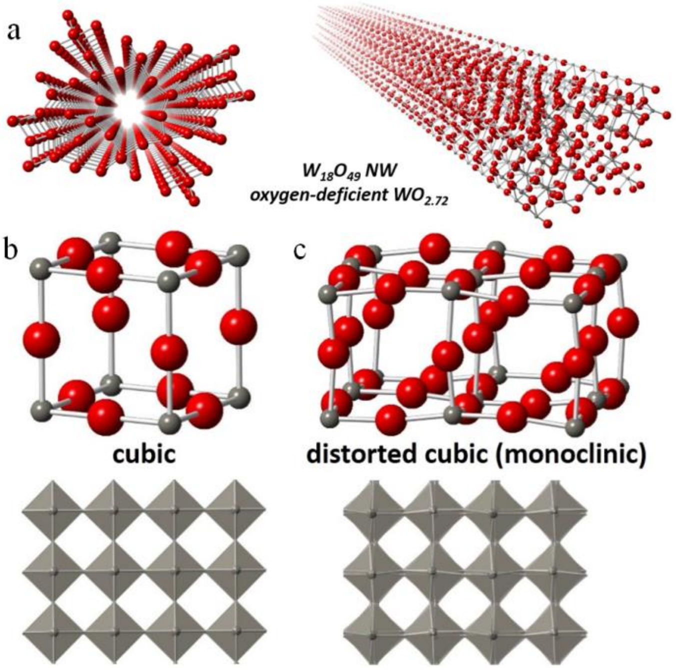

Supplementary Figure 8. (a) The crystalline structure of $W_{18}O_{49}$NW; Experimentally reported structures for cubic (b) and monoclinic (c) WO_3.

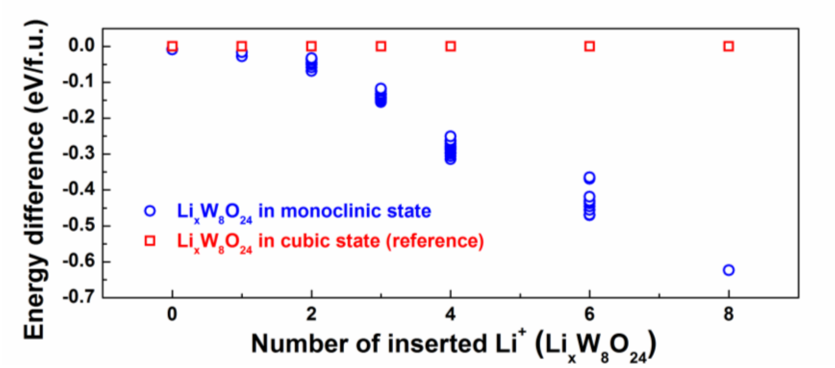

Supplementary Figure 9. The energy difference of lithiated WO_3 between cubic and monoclinic phases (cubic phase as reference state).

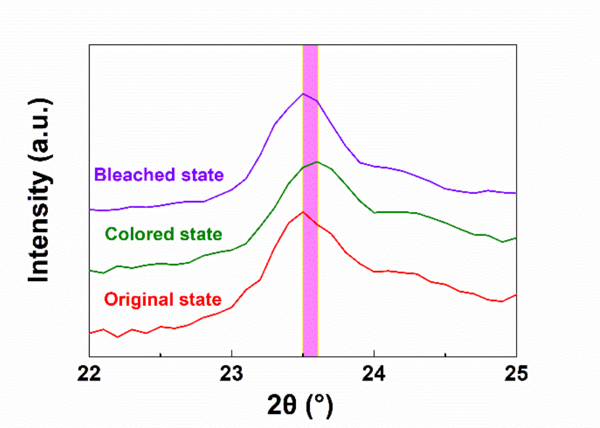

Supplementary Figure 10. *Ex-situ* GIXRDs of the dual-responsive film measured at the original, colored and bleached states, respectively.

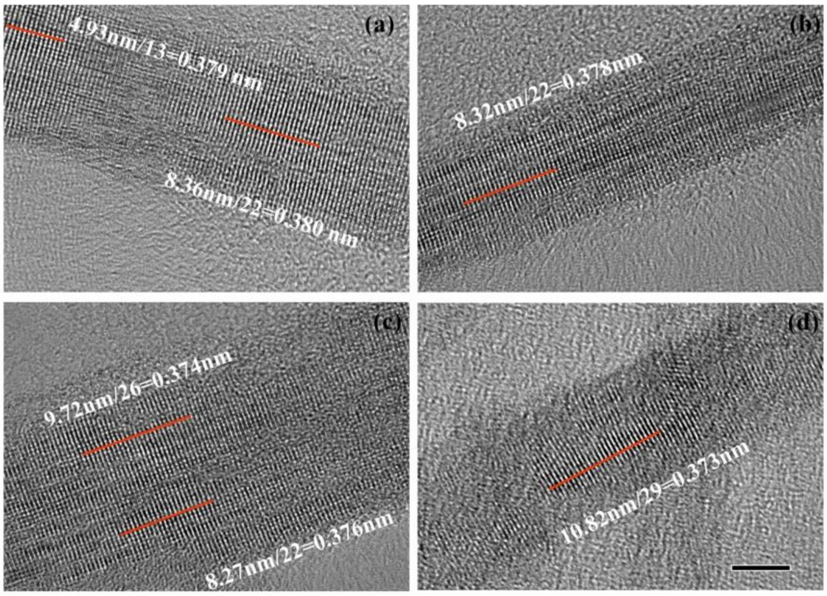

Supplementary Figure 11. HRTEM images of $W_{18}O_{49}$NWs measured at the original (a,b) and colored states (c,d) (Scale bar: 5 nm). The lattice spacings were calculated by the software of Gatan Digital Micrograph.

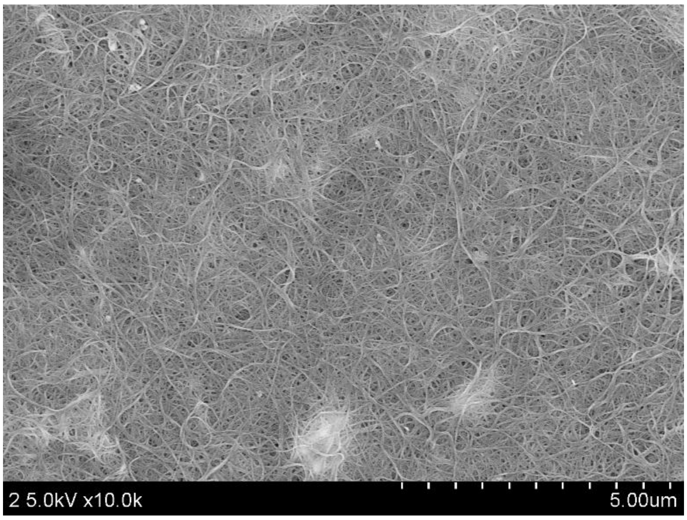

Supplementary Figure 12. SEM image of the SWCNT/$W_{18}O_{49}$NW composite film.

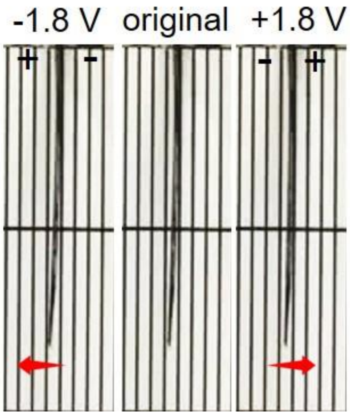

Supplementary Figure 13. Digital photographs of the SWCNTs based IPMC actuator under negative voltage (left), original state (middle) and positive voltage (right), respectively.

Interestingly, we found that the actuating direction of SWCNT actuators was opposite to that of the pseudocapacitive IPMC actuators. A possible reason concerns the electrolyte. For the assembly of air-operated SWCNT actuators, gel electrolytes (PMMA/PC/$LiClO_4$) must be used with the polymer as a mechanical framework. However, the polymer component in the gel electrolyte will block the efficient diffusion of ions, which leads to the decrease of ionic conductivity of the electrolyte. However, due to smaller ionic radius, Li ions (0.73 Å without solvation) can diffuse in gel polymer electrolyte and insert into the porous electrodes more easily and efficiently compared with ClO_4^- (2.40 Å without solvation) with much larger radius (http://www.wiredchemist.com/chemistry/data/thermochemical-radii-anions). Therefore, the cation (Li^+) diffusion and movement are dominant in the SWCNT actuators. Under this situation, when a voltage was applied, many Li^+ will move into negative electrodes quickly and lead to significant expansion of electrodes caused by the electrostatic repulsion among Li^+. Eventually, SWCNT actuators will be bent to positive electrodes which is opposite to the bending direction of the pseudocapacitive IPMC.

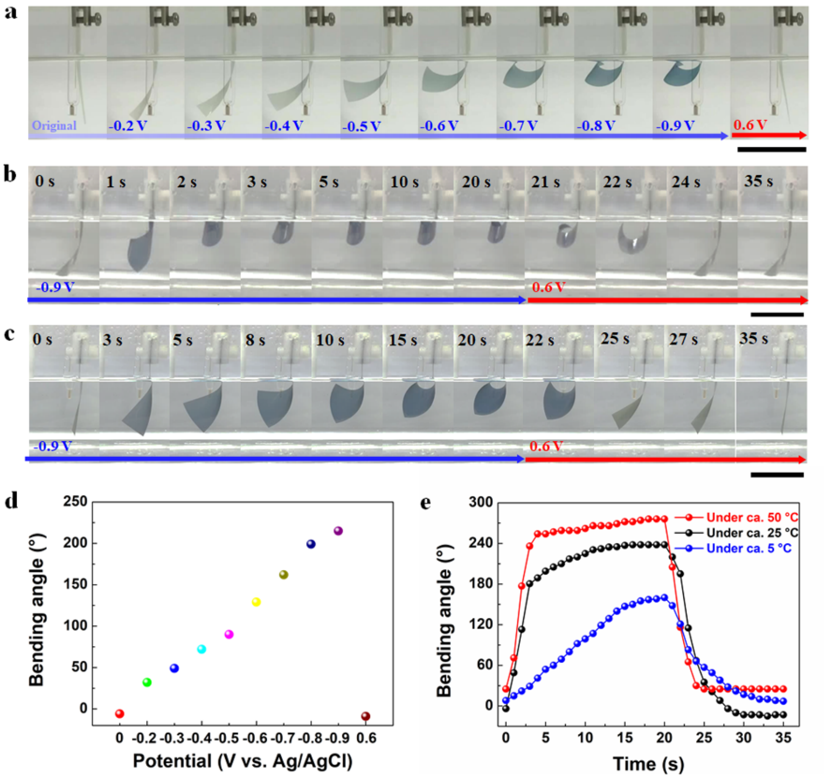

Supplementary Figure 14. Digital photographs of synchronous electrochromic/ actuating processes of the dual-responsive films under different applied potentials at room temperature (a), at elevated temperature (ca. 50 °C; b) and at lower temperature (ca. 5 °C; c), respectively; (d) Corresponding deformation angles of the dual-responsive film as function of the applied potentials; (e) The deformation angle responses of the dual-responsive film measured at +0.6 and −0.9 V bias under different temperatures. (Scale bars: 3 cm)

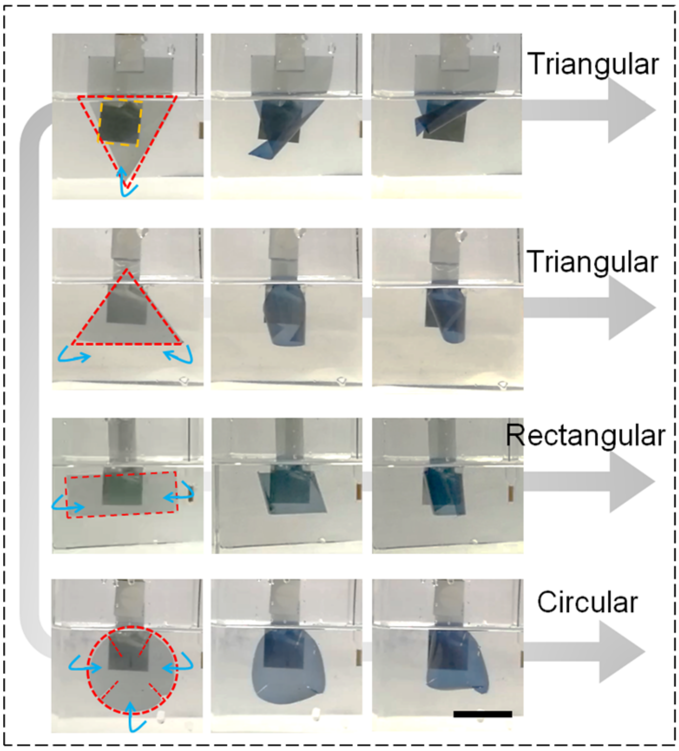

Supplementary Figure 15. Deformation responses of the dual-responsive films with different shapes (Scale bar: 2 cm). (The red dashed lines represent the active areas for deformation, i.e., the areas of dual-responsive films soaked in the electrolyte; The yellow dashed line represents the Pt counter electrode which is behind the dual-responsive film)

Supplementary Table 1. Comparison of the actuating performances between the dual-responsive film and conducting polymer-based actuators with bilayer configuration.

Electrode materials	Active ions	Bending angle (°)#	Curvature (cm^{-1})#	Strain (%)#	Response time (s)*	Ref.
PPy	EMI^+ $TFSA^-$	N.A.	N.A.	1.5	> 10	[1]
PPy	TEA^+ PF_6^-	N.A.	N.A.	~0.46	> 500	[2]
MWCNT/ PEDOT:PSS/PPy	TMA^+ Cl^-	~16.3	~0.142	N.A.	5	[3]
PPy/Graphene	Na^+ ClO_4^-	350	N.A.	0.7	32	[4]
PANI/Au	H^+ Cl^-	~180	N.A.	N.A.	< 5	[5]
PEDOT	Li^+ $TFSI^-$	56.4	~0.99	N.A.	50	[6]
PProDOT	Li^+ $TFSI^-$	53.9	~0.94	N.A.	50	[6]
**$W_{18}O_{49}$NWs**	**Li^+ ClO_4^-**	**238**	**1.22**	**1.81**	**< 5**	**This work**

Note: # Maximum/Best values. * Time required for maximum actuation.

Supplementary Table 2. Comparison of the actuating performances among different air-operating electrochemical actuators with trilayer configuration.

Electrode materials	Active ions	Displacement (mm)#	Curvature (cm^{-1})#	Strain (%)#	Actuating time (s)*	Ref.
PEDOT	EMI^+<br>$TFSI^-$	0.84	N.A.	0.9	0.0011	[7]
PEDOT:PSS	Li^+<br>$TFSI^-$	N.A.	~2.03	2.7	100	[8]
PPy	Na^+<br>$DBSA^-$	5.1	~0.562	N.A.	> 500	[9]
PPy	Li^+<br>$TFSI^-$	~12.5	0.656	0.83	< 2.5	[10]
CNT/PANI	EMI^+<br>BF_4^-	N.A.	~1.49	2.0	100	[11]
CNTǂ	EMI^+<br>BF_4^-	~0.28	~0.515	0.69	100	[11]
RGOǂ	BMI^+<br>BF_4^-	6.4	0.136	0.248	50	[12]
Graphdiyneǂ	BMI^+<br>BF_4^-	16	N.A.	0.77	5	[13]
NiONW@RGO/<br>MWCNTǂ	BMI^+<br>BF_4^-	15.0	~0.48	0.4	0.5	[14]
		~16.0	1.72	N.A.	50	
**SCNT/$W_{18}O_{49}$NWs**	**Li^+**<br>**ClO_4^-**	**1.83**	**0.141**	**0.12**	**1.4**	**This work**

Note: # Maximum/Best values. * Time required for maximum actuation. ǂ IPMC actuators.

Supplementary Note 1. Calculation of the bending curvature

As shown in the illustration for calculating curvature below, the bending angle θ can be expressed as following:

$$\theta = \frac{360 \times l}{2 \times \pi \times R} = \frac{180 \times l}{\pi R} \quad (1)$$

where r is the radius of curvature for the actuator, l is effective actuating length. Therefore, the curvature k can be calculated according to the equation (1):

$$k = \frac{1}{R} = \frac{\pi \theta}{180 \times l} \quad (2)$$

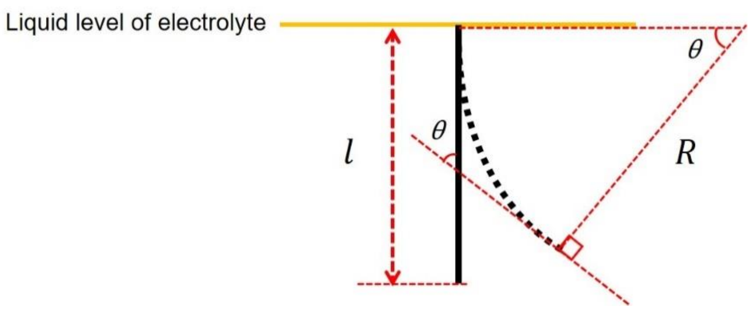

Supplementary Figure 16. The schematic illustration of calculation criteria for the bending curvature of dual-responsive films.

Supplementary Note 2. Calculation of the strain of active layer

Here, it is assumed that the BNN is a uniform active layer with thickness of 195 nm and is just consisting of pure $W_{18}O_{49}$NWs due to the much lower loading of AgNWs compared with $W_{18}O_{49}$NWs. Besides, the thickness of BNN active layer is assumed to be 0, because it is significantly smaller than that of PET substrate. Therefore, the strain was calculated according to following equation:

$$\varepsilon = \frac{\Delta l}{l} = \frac{l - 2\pi \times \frac{\theta}{360} \times (R - d)}{l} = 1 - \frac{\pi\theta(R - d)}{180l} = \frac{\pi\theta d}{180l}$$

where the d is the thickness of PET substrate.

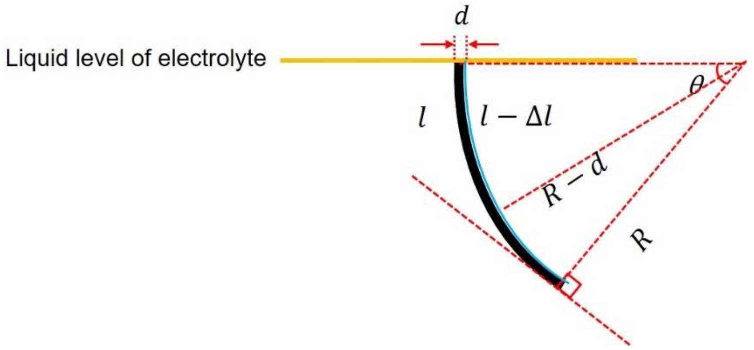

Supplementary Figure 17. The schematic illustration of measurement criteria for the strain of active layer.

Supplementary Note 3. Calculation of the bending curvature and strain of the pseudocapacitive IPMCs.

As shown in the illustration, the curvature *k* can be calculated according to the equation [15]:

$$k = \frac{1}{R} = \frac{2D}{L^2 + D^2}$$

where L is the effective length of the pseudocapacitive IPMCs.

The strain was calculated according to following equation [15]:

$$\varepsilon = \frac{\Delta L_1 - \Delta L_2}{L} = \frac{2DW}{L^2 + D^2}$$

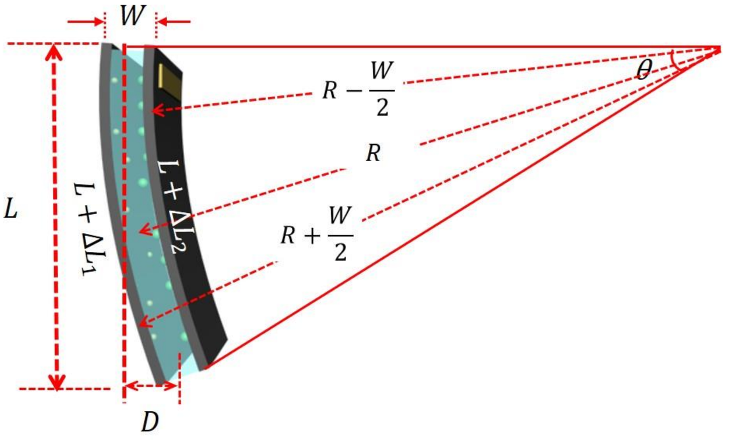

Supplementary Figure 18. The schematic illustration of measurement criteria for the bending curvature and strain of IPMC actuators during the deformation process.

Supplementary References:

[1] Ding, J. et al. Use of ionic liquids as electrolytes in electromechanical actuator systems based on inherently conducting polymers. *Chem. Mater.* **15**, 2392-2398 (2003).

[2] Warren, M. R. & Madden, J. D. A structural, electronic and electrochemical study of polypyrrole as a function of oxidation state. *Synth. Met.* **156**, 724-730 (2006).

[3] Kiefer, R. et al. Conducting polymer actuators formed on MWCNT and PEDOT-PSS conductive coatings. *Synth. Met.* **171**, 69-75 (2013).

[4] Liu, J. et al. A rationally-designed synergetic polypyrrole/graphene bilayer actuator. *J. Mater. Chem.* **22**, 4015-4020 (2012).

[5] Beregoi, M., Evanghelidis, A., Matei, E. & Enculescu, I. Polyaniline based microtubes as building-blocks for artificial muscle applications. *Sens. Actuators, B* **253**, 576-583 (2017).

[6] Gaihre, B., Ashraf, S., Spinks, G. M., Innis, P. C. & Wallace, G. G. Comparative displacement study of bilayer actuators comprising of conducting polymers, fabricated from polypyrrole, poly(3,4-ethylenedioxythiophene) or poly(3,4-propylenedioxythiophene), *Sens. Actuators, A* **193**, 48-53 (2013).

[7] Maziz, A. et al. Demonstrating kHz frequency actuation for conducting polymer microactuators. *Adv. Funct. Mater.* **24**, 4851-4859 (2014).

[8] Põldsalua, I. et al. Thin ink-jet printed trilayer actuators composed of PEDOT:PSS on interpenetrating polymer networks. *Sens. Actuators, B* **258**, 1072-1079 (2018).

[9] Ramasamy, M. S., Mahapatra, S. S. & Cho, J. W. Fabrication and characterization of dry conducting polymer actuator by vapor phase polymerization of polypyrrole. *J. Nanosci. Nanotechnol.* **14**, 7553-7557 (2014).

[10] Masurkar, N., Jamil, K. & Arava, L. M. R. Environmental effects on the polypyrrole tri-layer actuator. *Actuators* **6**, 17 (2017).

[11] Sugino, T., Kiyohara, K., Takeuchi, I., Mukai, K. & Asaka, K. Actuator properties of the complexes composed by carbon nanotube and ionic liquid: The effects of additives. *Sens. Actuators, B* **141**, 179-186 (2009).

[12] Lu, L. et al. Highly stable air working bimorph actuator based on a graphene nanosheet/carbon nanotube hybrid electrode. *Adv. Mater.* **24**, 4317-4321 (2012).

[13] Lu, C. et al. High-performance graphdiyne-based electrochemical actuators. *Nat. Commun.* **9**, 752 (2018).

[14] Wu, G. et al. An interface nanostructured array guided high performance electrochemical actuator. *J. Mater. Chem. A* **2**, 16836-16841 (2014).

[15] Sugino, T., Kiyohara, K., Takeuchi, I., Mukai, K. & Asaka, K. Actuator properties of the complexes composed by carbon nanotube and ionic liquid: The effects of additives. *Sens. Actuators, B* **141**, 179-186 (2009).
