# Supplementary material for: Lattice-contraction triggered synchronous electrochromic actuator
Source: Nat Commun. 2018 Nov 15;9:4798. doi: 10.1038/s41467-018-07241-7 (PMC6237766; doi:10.1038/s41467-018-07241-7)
Supplement: Supplementary file 3 — Description of Additional Supplementary Files [file 41467_2018_7241_MOESM3_ESM.pdf]

Description of Additional Supplementary Files

File Name: Supplementary Movie 1
Description: The dual-responsive film showed great synchronism and excellent electrochromic and actuating performances in 1 M LiClO4/propylene carbonate electrolyte.

File Name: Supplementary Movie 2
Description: The pseudocapacitive IPMC actuator bent to the left and right under the constant voltage of 1.8 V and −1.8 V, respectively.
